# Social Determinants of Long Lasting Insecticidal Hammock-Use Among the Ra-Glai Ethnic Minority in Vietnam: Implications for Forest Malaria Control

**DOI:** 10.1371/journal.pone.0029991

**Published:** 2012-01-12

**Authors:** Koen Peeters Grietens, Xa Nguyen Xuan, Joan Muela Ribera, Thang Ngo Duc, Wim van Bortel, Nhat Truong Ba, Ky Pham Van, Hung Le Xuan, Umberto D'Alessandro, Annette Erhart

**Affiliations:** 1 Department of Public Health, Institute of Tropical Medicine, Antwerp, Belgium; 2 PASS International, Tessenderlo, Belgium; 3 National Institute for Malariology, Parasitology and Entomology, Hanoi, Vietnam; 4 Provincial Malaria Station, Phan Rang, Vietnam; 5 Medical Research Council Unit, Basse, The Gambia; 6 Department of Biomedical Sciences, Institute of Tropical Medicine, Antwerp, Belgium; London School of Hygiene and Tropical Medicine, United Kingdom

## Abstract

**Background:**

Long-lasting insecticidal hammocks (LLIHs) are being evaluated as an additional malaria prevention tool in settings where standard control strategies have a limited impact. This is the case among the Ra-glai ethnic minority communities of Ninh Thuan, one of the forested and mountainous provinces of Central Vietnam where malaria morbidity persist due to the sylvatic nature of the main malaria vector *An. dirus* and the dependence of the population on the forest for subsistence - as is the case for many impoverished ethnic minorities in Southeast Asia.

**Methods:**

A social science study was carried out ancillary to a community-based cluster randomized trial on the effectiveness of LLIHs to control forest malaria. The social science research strategy consisted of a mixed methods study triangulating qualitative data from focused ethnography and quantitative data collected during a malariometric cross-sectional survey on a random sample of 2,045 study participants.

**Results:**

To meet work requirements during the labor intensive malaria transmission and rainy season, Ra-glai slash and burn farmers combine living in government supported villages along the road with a second home at their fields located in the forest. LLIH use was evaluated in both locations. During daytime, LLIH use at village level was reported by 69.3% of all respondents, and in forest fields this was 73.2%. In the evening, 54.1% used the LLIHs in the villages, while at the fields this was 20.7%. At night, LLIH use was minimal, regardless of the location (village 4.4%; forest 6.4%).

**Discussion:**

Despite the free distribution of insecticide-treated nets (ITNs) and LLIHs, around half the local population remains largely unprotected when sleeping in their forest plot huts. In order to tackle forest malaria more effectively, control policies should explicitly target forest fields where ethnic minority farmers are more vulnerable to malaria.

## Introduction

Hammocks protected by insecticide-treated nets (ITHNs) or by long-lasting insecticidal nets (LLIHs) have been recommended as additional malaria prevention tools in settings where standard control strategies have a limited impact [1], [2]. While bed nets are less effective when the vector bites outdoors and/or early in the evening when people are still active, indoor residual spraying (IRS) faces similar problems when the vector does not rest indoors [3] or when house structures are open [4]. Hammock nets are therefore expected to provide extra protection in the evening when people are not yet sleeping under bed nets and in conditions where bed nets are not likely to be used, i.e. during forest activities such as hunting, logging and sleeping at forest plot huts during harvests.

The growing need for additional protection tools is directly related to the progressive confinement of malaria to specific areas and risk populations. This is currently the case in Vietnam where malaria now mainly affects poor ethnic minorities in remote areas, forest workers, and migrants [5]–[11]. The difficulty of controlling forest malaria with the classical vector control interventions (ITNs and IRS) has also been shown in other regions of Southeast Asia [12]–[16] and has prompted the need for tailoring and evaluating additional tools better adapted to these specific settings.

As an additional control tool, hammock nets are likely to be more cost-effective and user-friendly than certain other options such as mosquito coils, which need to be replaced nightly, and repellents which also require continuous application. Furthermore, the presence of LLIHs could contribute to a cumulative insecticidal effect as previously reported in West Africa [1].

In terms of efficacy, the few studies carried out so far on hammock nets showed promising results. Hougard et al. [1] determined the efficacy of LLIHs under well-controlled conditions in experimental huts in Benin. The repellent effect of the hammocks significantly reduced the number of mosquitoes entering the hut, and the high mosquito mortality further indicated that a mass killing effect may occur if their use were widespread. In Suriname [17], mortality among mosquitoes leaving a hut with ITHNs was 58% as compared to 27% in huts without hammock nets. Still in Suriname, between 1989 and 1991, the use of ITHNs drastically reduced malaria prevalence after 36 months of use in Southern Amerindian villages (J. Voorham, unpublished data, in [17]). Similarly, in the Venezuelan Amazon region inhabited by the Yanomami, ITHNs prevented 56% of new malaria cases during a 2-year follow-up [18]. In Cambodia, an entomological study showed that, although LLIHs did not induce full protection against malaria vectors, they could prove effective in protecting forest workers and villagers before sleeping and be a valuable additional tool in eliminating artemisinin-resistant malaria in the region [19]. The effectiveness of the LLIH design used in the Cambodian study was evaluated in a large cluster randomized trial in South-Central Vietnam (hereafter “LLIH-trial”), where a significant reduction in malaria prevalence and incidence was observed over the 2-year study period [20]. While the LLIH-trial reported estimates on the overall hammock net use by study participants, the epidemiological data were unable to provide an in-depth understanding of the factors influencing LLIH-use. An ancillary social science study was conducted to fill this gap and provide comprehensive data on LLIH-use patterns, their adequacy in the local context, acceptability and related social and contextual determinants of LLIH-use. The results of this study are reported here.

## Methods

### Study Site and Population

The study area, covering 30 villages (Bac Ai and Ninh Son districts) situated in the hilly and forested part of Ninh Thuan province (South-Central Vietnam), is traditionally inhabited by the Ra-glai ethnic minority which represents 86% of the study population [21]. The Ra-glai are a largely impoverished ethnic minority, almost exclusively dedicated to small-scale subsistence slash and burn agriculture in fields located in the surrounding forests. According to a survey carried out in 2003, 80% of the active population can be categorized as ‘forest worker’, 99.5% of which participate in slash and burn agriculture, occasionally combined with hunting, gathering of forest products and logging [21]. The Ra-glai's heavy dependence on the forest for subsistence places them at greater risk for malaria infection, a risk that is further increased by staying overnight in the forest [21], [22].

**Figure 1:**
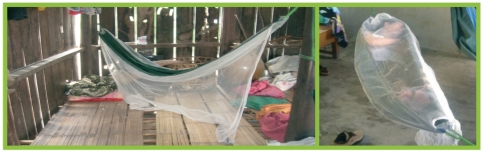
LLIH used for the trial.

Malaria transmission in the region is perennial with 2 peaks at the start and at the end of the rainy season (May–June and October–November). The main vector is *An. dirus sensu stricto*, a sylvatic and highly anthropophilic species whose exophagy and exophily, as well as early biting habit, challenge the impact of standard interventions such as IRS or ITNs [3], [23]. Before the start of the LLIH-trial, in 2003, the overall parasite rate was 13% (up to 40% in some villages) and the prevalence of antimalarial antibodies 37% (up to 75% in certain villages) [21]. Malaria control is based on early diagnosis and treatment with artemisinin-based combination therapy and on the provision of ITNs distributed free-of-charge by the national malaria control program. The LLIHs distributed during the trial consisted of a green nylon hammock covered with Olyset netting double as wide as the hammock itself; half of which was sewn onto the back of the hammock and the other half consisting of a free flap to cover the person lying inside [17], [20] (Figure 1).

### Research Strategy

The research strategy consisted of a mixed methods research design, triangulating qualitative data from focused ethnography and quantitative data collected during a large scale cross-sectional survey carried out in the framework of the LLIH-trial. This methodological triangulation was preferred in order to limit bias and build upon the strengths of the respective methods [24]–[26]. Qualitative data were gathered first for independent analysis and additionally as a preparatory strand to elaborate questions to include in the cross-sectional malariometric survey. Data proceeding from the qualitative study are reflected in the context analysis of the results section while survey data (quantitative) are summarized in tables and figures.

### Qualitative Data

A focused ethnographic study was carried out during three field stays of approximately one month each between July 2005 and September 2006, in the intervention arm of the LLIH-trial. During fieldwork, 12 villages were purposefully selected for their theoretical relevance [25], representing seven communes distributed in the two study districts [20]. Selection criteria included malaria incidence, accessibility, socio-economic level of the population, and availability of health services.

#### Data collection

Qualitative data collection techniques consisted of participant observation, interviews, and group discussions. During participant observation, the researchers participated in everyday activities, observing events in their usual context and carrying out reiterated informal conversations and interviews in order to build up confidence with informants and acquire an in-depth understanding of more sensitive subjects such as adherence to official malaria control policies, including LLIH and ITN use. Observation was a key aspect of the methodology since it is a respondent independent technique and therefore extremely valid to overcome the bias often inherent in self-reporting techniques [25] such as pre-coded standardized questionnaires.

Interviews were carried out after respondents' verbal consent and, when applicable, recorded and transcribed. In those cases that the interviewer(s) considered that recording or taking notes in the presence of the respondent was not appropriate (due to the sensitive nature of the subjects discussed, the required informality of the interview, the respondents' preferences or other limitations) the content of the conversation was not recorded but written down immediately after the interview. Semi-structured interviews and informal conversations were held repeatedly with all included households during fieldwork to reduce social desirability and acquiescence bias.

Only informal group discussions were held since the inherent cultural respect for social hierarchy led to high response bias in formal focus group discussions.

#### Sampling

Multiple purposive sampling techniques [27] were used throughout the study. Based on the principle of gradual selection, informants were theoretically selected (in accordance with emerging results/theory) and categorized in relation to relevant criteria (such as gender, age, locality, forest activities, previous experience of malaria, use of preventive measures, etc.). In addition, critical cases were continuously selected to allow for maximum variation and internal diversity. Snowball sampling techniques (sampling using participants to identify additional cases) were used in order to increase respondents' trust and consequently reduce response bias (i.e. new respondents can be expected to be more confident in the researchers' trustworthiness after an acquaintance's referral). All qualitative techniques were applied in all selected study villages until saturation (no new information could be generated).

### Quantitative data

In order to confirm and quantify research hypotheses generated by the qualitative approach, a total of 20 closed-ended questions were included in the standardized pre-coded questionnaire of the malariometric survey carried out in the framework of the LLIH trial in December 2006 (hereafter “2006-survey”) [20]. The questionnaire was administrated to all survey participants in the intervention arm and was specifically related to the Ra-glai house-settlement system, forest activities, LLIH-use, sleeping habits and knowledge and practices related to malaria exposure.

### Mosquito Collections

Ancillary to epidemiological and anthropological data collections, five entomological surveys were carried out between November 2004 and December 2006 –the detailed methodology and results of which are published elsewhere [28]. Briefly, human outdoor landing collections were done inside the villages and near forest shelters for eight nights per survey in each location (eight villages). Mosquitoes were collected from 18.00h until 06.00h, stored by hour and morphologically identified in the field using a standardized key for medically important anophelines in Southeast Asia. For the purpose of the present paper, the results on cumulative biting activities of the three main vectors, *Anopheles dirus s.l.*, *An. minimus s.l.* and *An. maculatus* were pooled at villages and at forest fields from 18h00 to 6h00 and were compared to the corresponding human activities as determined in the 2006-survey (sleeping times were calculated based on the percentages of people stating to be asleep at a certain time and location).

### Data analysis

#### Qualitative data

In accordance with the research strategy, data gathering and analysis were concurrent and data analysis was a continuous, flexible and iterative process. Preliminary data -collected through different techniques- were intermittently analyzed in the field (sequential analysis) after which further research was conducted confirming or refuting temporary results through constant validity checks until saturation was reached and the data could be theoretically supported. Raw data were processed in their textual form and coded to generate and/or identify analytical categories or themes for further analysis. Analytic induction, involving the iterative testing of theoretical ideas, was used to refine and categorize themes grounded [29] in the data while emerging (and absent) themes were additionally evaluated in dialogue with existing social science theory resulting in an analytical framework that was then systematically applied to all the data. The systemization and analysis were carried out with NVivo Qualitative Data Analysis software (QSR International Pty Ltd. Cardigan UK).

#### Quantitative data

The 2006-survey data were double entered and cleaned in Epi Info 6.04 (CDC, Atlanta; WHO, Geneva 1996), and analyzed with STATA 9.0 software (Stata Corp., College Station, TX). Descriptive statistics were computed using the “svy” command in STATA, in order to take into account the survey characteristics.

### Ethical considerations

The study protocol was approved by the ethics committees of the Institute of Tropical Medicine (Antwerp, Belgium) and the National Institute of Malariology, Parasitology and Entomology (Hanoi, Vietnam) as well as by the Vietnamese Ministry of Health. During the ethnographic data collection, all interviewers followed the Code of Ethics of the American Anthropological Association [30]. All interviewees were informed in their local language about project goals, the topic and type of questions, their right to refuse being interviewed, to interrupt the conversation at any time, and to withdraw any given information during or after the interview, Verbal consent was preferred, since the act of signing one's name when providing information during informal conversations could be a potential reason for mistrust [31]. All interviews were carried out by the principal investigator and a co-researcher/witness of the consent procedure. For the malariometric survey, the research procedures were disclosed to all participants (community leaders and local authorities were witnesses) at the time of the census, and oral informed consent was sought from them or their legal representatives. It was estimated that the procedure of verbal consent would be sufficient as people living in the study villages could choose on whether or not to use the intervention (LLIH). In addition, the study procedures -i.e. the identification of malaria infections at village and health facility level- including the cross-sectional surveys, were within the activities carried out by government authorities for the purpose of malaria control.

## Results

### Residence patterns and seasonality

Qualitative data showed that to meet work requirements during the labor intensive malaria transmission and rainy season, Ra-glai slash and burn farmers combine living in government supported villages along the road with a second home or shelter at their slash and burn fields located in the forest. The exact amount of time spent in either residence is difficult to determine with certainty due to the strong response bias among the Ra-glai who, in accordance with official policies, are expected to sleep exclusively in the government provided houses. Qualitative research showed, however, that during the rainy season and especially during harvest times, the large majority of Ra-glai families spend nights at their fields because, among other factors, they lose less time traveling between their houses and fields and are able to safeguard their harvests from rodents, cattle and other animals. While the most important activity during the rainy season is slash and burn farming on fields located in the forest, during the dry season the farming workload diminishes and activities shift to the villages, allowing farmers to rest after harvests, buy and sell harvested products and engage in other economic activities such as cattle herding, hunting, gathering, logging, and wage work.

### LLIH use and human activity patterns

The estimation of LLIH-use in village homes was done on all survey participants (2,045 individuals), and in plot huts at forest fields on a sub-sample of 280 respondents that stated to have slept at their fields in the month prior to the survey. This sub-group had a sex ratio similar to the total survey population but the age distribution showed a deficit in children (<10 years old) compensated by an excess of elderly (>50 y).

Overall LLIH-use was high since 92.8% of the respondents used them at their village homes (only 7.2% reported never using LLIH) and 82.9% when staying at forest fields (Table 1). LLIHs were primarily used to rest during the day, i.e. 69.3% of village respondents and 73.2% among farmers that slept at forest fields. For 36.8% of village respondents and 59.3% of farmers at forest fields this was the only time LLIHs were used. In the evening, LLIHs were used by 54.1% of village respondents and 20.7% of farmers at forest fields. LLIH use at night was generally low (village 4.4%; forest 6.4%). Overall, LLIHs were used at a time relevant for malaria control (in the evening *and/or* at night) by 56.0% (n = 1146/2045) of village respondents and 23.5% (n = 66/280) of farmers at forest fields.

**Table 1:** Reported LLIH use according to residence (Dec. 2006 survey).

LLIH use in villages (N = 2,045)	N	%
Never	147	7.2
During the day (in possible combination with evening and/or night)	1417	69.3
Exclusively during the day	752	36.8
In the evening (in possible combination with day and/or night)	1106	54.1
At night (in possible combination with day and/or evening)	91	4.4

LLIH use at forest fields (N = 280)		
Never	48	17.1
During the day (in possible combination with evening and/or night)	205	73.2
Exclusively during the day	166	59.3
In the evening (in possible combination with day and/or night)	58	20.7
At night (in possible combination with day and/or evening)	18	6.4

**Figure 2:**
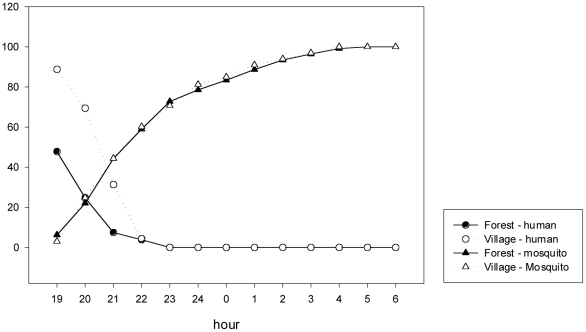
Biting curves (cumulative percentages) of the three main vectors, *Anopheles dirus s.l.*, *An. minimus s.l.* and *An. maculatus* pooled compared to activity curves (not asleep, cumulative percentages) by hour and location (village/forest).

The timing of LLIH-use can be understood in relation to local human activity patterns. Qualitative data show that, at their fields, Ra-glai farmers wake up and start preparing food and daily farming activities before daybreak (around 04.00h) and stop working before sunset at around 17.00/17.30h. Dinner is usually early, at around 18.00–18.30h, and after sunset (+/−18.30h) all social activities take place indoors since there is no electricity and because the daily workload is exhausting during the rainy season. Human activity patterns estimated in the 2006-survey show that approximately half (52%) of Ra-glai respondents report to be asleep by 19.00h, only 24.5% were still awake after 20.00h, and by 21.00h almost everybody (92%) was asleep (Figure 2). In the villages, the Ra-glai go to bed later as electricity allows people to engage in leisure activities such as watching TV, singing Karaoke and having drinks at small bars. Most respondents were awake until 20.00h; at 21.00h, this was still 31% and only 4.3% was awake after 22.00h (Figure 2). When relating the human activity patterns to mosquito biting times, the *Anopheles* vectors had the highest biting activity in the evening, with 6% of the bites by 19.00h, 25% by 20.00h and 50% before 22.00h. No difference in cumulative percentages of mosquito bites was observed between the forest and the villages (Figure 2). Correlating human/mosquito activity patterns (Figure 2) with the proportion of people protected by either LLIH and/or ITNs (Table 2), it appears that at the forest fields, local farmers are exposed to mosquito bites mainly due to low ITN use. Indeed, approximately half (52%) of Ra-glai respondents were asleep by 19.00h but only 58% would regularly be protected by ITNs. Furthermore, among the fraction of people staying out later at the forest fields only about 20% would be using LLIHs. Comparatively, at villages, both people staying out late and those sleeping were more likely to be protected, respectively by LLIHs (56%) and ITNs (92%).

**Table 2:** Use of malaria preventive measures by residence (2006 survey).

	ITN		LLIH (evening/night)	
	n/N	%	n/N	%
**Villages**	1883/2045	92.1	1146/2045	56.1
**Forest Field**	162/280	57.9	66/280	23.6

### Traditional sleep provisions

The Ra-glai traditionally sleep on the wooden or bamboo floor of their mostly stilted houses. In accordance with the characteristics of farming activities, 89.01% of households go to their forest fields together, including children and elderly [22]. Sleep provisions at the fields are therefore mostly communal –i.e. provided for the entire family. Qualitative research points out that a perceived disadvantage of the individual LLIHs was that they do not permit men and women, or women and children (with the possible exception of small infants) to sleep together. Nevertheless, there are certain important exceptions. For one, hammocks are used as cradles and children rest in them during the day and evening, especially in the time span before other household members go to sleep. Also, while couples sleep with small children, older children and the elderly (especially when widowed) might sleep separately using hammocks, provided there is available space to hang the hammock.

### Mobility

The mentioned double residence system (village/forest fields) further leads to complex mobility and sleeping patterns that generally affect the use of preventive measures (Table 2). While the entire family moves together for longer periods of time, one or more individual household member(s) might be required to sleep sporadically at the other location (field/village), examples of which can include children attending school, adults bringing products to the market, buying provisions at local shops or visiting relatives. In these cases, he/she can opt to take the ITN and/or LLIH and leave other household members unprotected or sleep unprotected while away.

### Irregular forest overnights

While during the rainy season the Ra-glai are almost exclusively dedicated to farming, other activities (hunting, gathering forest products, logging) carried out mostly in the dry season entail overnights deep in the rain forest for which LLIHs could be a good personal malaria prevention tool. Currently, however, qualitative data suggest that hunters and loggers sleep at clearings in the forest or on rocks next to rivers and carry blankets rather than bed nets or hammocks, as the blankets are believed to be sufficient in protecting against mosquitoes and additionally keep people warm.

## Discussion

Despite the relatively common use of LLIHs, both during the day and in the evening in government supported villages, hammock nets were less used where and when exposure was the highest: in the evenings and at night at forest fields where farmers mostly stay during the rainy season corresponding to the peak of malaria transmission. Indeed, at forest fields, farmers are more likely to be exposed due to the sylvatic nature of *An. dirus* and the general low uptake of prevention measures (LLIH 24% and ITN use 58%). Comparatively, at villages, the uptake of both ITNs and LLIHs was higher than in forest fields. Thus LLIHs were likely to have a protective effect at village level since they offer protection to about half of the population that would otherwise be completely exposed during evening activities. Nevertheless, these findings also highlight that, even with free of charge LLIH distribution but without specific prevention malaria control policies targeting fields, LLIH use at forest fields at times relevant for malaria prevention remains low (20.7% evening, 6.4% at night). Quantitative results concerning LLIH and ITN use, especially those related to overnights in the forest, are difficult to interpret due to social desirability bias in response to questions concerning overnights at forest fields and adherence to public health interventions in a context where impoverished ethnic minority farmers are under direct and indirect acculturation pressure. This is apparent in the low number of participants actually reporting to sleep at forest fields while this is common practice, representing a limitation of the current study despite the fact that qualitative research did not uncover any relation between the observed social desirability bias and LLIH-use. For the same reason, the use of LLIHs during hunting and logging could not be properly quantified, as those activities are illegal, leading to a general reticence to discuss them.

The low use of preventive measures at fields can be related to the unexpected double residence system among the Ra-glai that requires protective measures both in government-supported villages along the road and in shelters at fields. Though LLIHs were distributed to all residents (≥10 years old) in the intervention clusters, accounting for 70% coverage [20], they would either have to be continuously transported between villages and fields or, more practically, simply left in the place of preference, generally the village -presumably due to the better housing conditions and the longer leisure time (hammocks were mostly used for resting and possible leisure activities and not to sleep in). The same applies to ITNs which, with a median coverage of 2.5 persons per net in the study area [22], also have to be transported between villages and fields and have the additional difficulty of leaving household members exposed when required to sleep in different places. This difficulty –together with the low perception of malaria risk in the same population as previously described [32]– is reflected in the low use of both ITNs and LLIHs at fields as compared to villages.

The higher use of LLIHs in villages becomes clear when understanding that there is hardly any time to rest in the evening at the fields while in the villages social activities in the evening last longer, especially in periods when the workload is lighter (i.e. harvesting at fields versus trading products in villages). The low use of LLIHs at night can be attributed to two main factors. First, LLIHs provide protection for the individual but do not allow married couples or mothers and children to sleep together. Second, Ra-glai farmers traditionally sleep on wooden or bamboo floors and not in hammocks (similar observations have been made by the authors in other regions of Vietnam and Cambodia). In other contexts, certain ethnic groups do traditionally sleep in hammocks, such as the Yanomami in Venezuela, where the hammocks are arranged around the fire in a single large circular house [19] and in the Upper-Maroni Amazon of French Guyana, where people reportedly use hammocks to sleep with non-impregnated hammock nets [33]. Despite not conforming to traditional sleeping arrangements, LLIHs among the Ra-glai could still prove to be a promising malaria control tool especially for people sleeping individually and irregularly in the forest, such as during the dry season's activities.

Interestingly, despite the relatively low LLIH use in the evening and at night at the fields, a significantly stronger decrease in the incidence of malaria attacks and the prevalence of infection was observed in the intervention compared to control clusters during the 2-year trial period [20]. The protective effect of the LLIH can therefore probably be attributed to a better protection in the villages in the evening, which is further accentuated by the fact that certain endemic villages are situated at the fringe of the forest, blurring the difference in exposure between villages and forest fields. On the other hand, despite the absence of significant interaction between intervention group and age [20], the additional protective effect provided by LLIHs might have been stronger in younger age groups and children (A. Erhart, personal communication). This would be consistent with the fact that LLIH use was often reserved for children: as cradles for babies and to rest in for children.

### Implications for forest malaria control in Southeast Asia

Even with additional LLIHs and free of charge ITN distribution, the low uptake of preventive measures at farmers' fields represents one of the main bottlenecks for effective forest malaria control and elimination in areas where populations are dedicated to slash and burn agriculture. Research into such settings and populations, that represent social and cultural characteristics other than those of majority society, is not only relevant for the further reduction of the local malaria burden but also for long term malaria elimination goals as these groups may constitute a residual silent parasite reservoir as earlier surveys have shown high proportions of asymptomatic infections [11], [21], [22]. Sleeping at fields seems to be a general characteristic and requirement of slash and burn agriculture in Southeast Asia (and has also been observed by the authors for other forms of agriculture in Sub Saharan Africa). It might be further hypothesized that, whether stimulated by government policies to settle in fixed clusters or drawn to main roads and infrastructure because of the commodities they offer, subsistence slash and burn farmers cannot sustain themselves in larger villages without resorting to sleeping at fields. Consequently, with increasing walking distance between villages and forest fields, people are more likely to stay overnight at their forest plot huts. Nonetheless, epidemiological indicators refer to village homes only since no questions are asked about possible double residences; the number of ITNs per household is calculated based on one single residence; and, standard malaria control programs directly target villages and not the farmers' fields where they are more at risk and where they spend a considerable amount of time, including nights, with their families to meet work requirements during the rainy season. And, unless poverty levels change drastically, they can be expected to continue to do so, even when aware of the enhanced malaria risk. Additional measures therefore need to be taken, such as malaria control policies specifically targeting forest fields to protect the most vulnerable populations in economically expanding Southeast Asian societies not only to reduce the local malaria burden but also, in the light of malaria elimination, to minimize the risk of spreading malaria to other areas where transmission had virtually ceased.
